# Congenital Heart Disease and the Risk of Cancer: An Update on the Genetic Etiology, Radiation Exposure Damage, and Future Research Strategies

**DOI:** 10.3390/jcdd9080245

**Published:** 2022-08-01

**Authors:** Jonica Campolo, Giuseppe Annoni, Marzia Giaccardi, Maria Grazia Andreassi

**Affiliations:** 1 CNR Institute of Clinical Physiology, ASST Grande Ospedale Metropolitano Niguarda, 20162 Milan, Italy; campolo@ifc.cnr.it; 2 Pediatric Cardiology, ASST Grande Ospedale Metropolitano Niguarda, 20162 Milan, Italy; giuseppealberto.annoni@ospedaleniguarda.it; 3 Electrophysiology Unit, Department of Internal Medicine, Santa Maria Annunziata Hospital, 50012 Florence, Italy; marzia.giaccardi@uslcentro.toscana.it; 4 CNR Institute of Clinical Physology, 56125 Pisa, Italy

**Keywords:** congenital heart disease, cancer, genetics, ionizing radiation

## Abstract

Epidemiological studies have shown an increased prevalence of cancer in patients with congenital heart disease (CHD) as compared with the general population. The underlying risk factors for the acquired cancer risk remain poorly understood, and shared genetic anomalies and cumulative radiation exposure from repeated imaging and catheterization procedures may be contributing factors. In the present review, we provide an update on the most recent literature regarding the associations between CHD and cancer, with a particular focus on genetic etiology and radiation exposure from medical procedures. The current evidence indicates that children with CHD may be a high-risk population, already having the first genetic “hit”, and, consequently, may have increased sensitivity to ionizing radiation from birth or earlier. Future research strategies integrating biological and molecular measures are also discussed in this article.

## 1. Introduction

Congenital heart disease (CHD) is the most common birth defect in humans, affecting approximately 1% of all births [1].

Advances in pediatric cardiovascular surgery and cardiac interventional catheterization have increased the survival of patients with CHD (>90% reach adolescence and adult life), even those with complex defects [2].

Children with congenital defects have a significantly higher risk of developing some type of cancer, including leukemia, tumors of the central nervous system, tumors of the sympathetic nervous system, and soft tissue sarcomas [3,4,5,6,7,8]. Cancer risk is especially increased in CHD children with complex defects [9,10].

At the present, it is critical to understand the burden of cancer in this patient population, as well as the identification of contributing risk factors. However, the etiology of cancer in patients with CHD remains largely unknown.

In the present review, we provide an update on the most recent literature regarding the associations between CHD and cancer, with a particular focus on genetic etiology and radiation exposure from medical procedures. The current challenges and future research strategies integrating biological and molecular measures are also discussed in this article.

## 2. Research Design and Methods

Published data useful for this paper were identified by searching in PubMed, Scopus, and Google Scholar until June 2022. The research was performed using the following search terms: “congenital heart disease” OR “congenital heart defect” AND “cancer” OR “ionizing radiation”. We considered articles written in English and available to the reader. Additionally, relevant papers were also manually screened for additional suitable studies. The review included only studies assessing patients with CHD, and findings were presented using a narrative style to allow for wide readability on the main findings.

## 3. Cancer Risk in CHD Patients

Previous studies have reported an association between CHD and cancer (Table 1), although it remains conflicting and inconclusive.

Since some genetic diseases—in particular, disorders caused by chromosomal alterations—may specifically affect this risk [8,9], some epidemiological studies excluded CHD patients with associated genetic syndrome from their analyses, as described below.

In the register-based Danish study, the overall cancer risk was not significantly elevated among patients with CHD after excluding those with Down syndrome or Fanconi anemia, as compared to the general population [11].

However, other studies suggested a potentially elevated cancer risk among patients with a diagnosis of CHD [7,12,13,14], both in children and adults.

In particular, Collins et al. [9] reported elevated total childhood cancer and lymphoma risks in CHD patients without chromosomal anomalies, coming from a Californian population-based cohort study.

A population-based study on 31,961 CHD patients from Taiwan showed an increased risk of cancer compared with the general population (standardized incidence ratio, 1.45; 95% confidence interval, 1.25–1.67) [12]. In this study, the CHD cohort also included patients with chromosomal anomalies.

Mandalenakis et al. found that patients with CHD had increased risk of developing cancer (HR  =  2.24, 95% CI, 2.01–2.48) as compared to healthy matched controls, from the Swedish Patient Register, and the risk was significantly higher among patients with CHD from the most recent birth cohort having developed cancer by age 18 years or younger [13]. This study was based only on the administrative data without clear clinical information on the associated genetic syndrome in the CHD cohort.

CHD patients in Quebec have 1.6 to 2 times higher cancer prevalence than that in the general Canadian population [14]. The increased risk in their study was not related to Down syndrome alone, as those patients made up only a very small proportion of the CHD study cohort.

Recently, a large population-based cohort study by Kampitsi et al. [10] reported a 64% increased risk of lymphoma and more than 300% of hepatoblastoma, respectively. The risk of lymphoma was particularly elevated in children with complex CHD and that of leukemia in patients with Down syndrome [10]. Additionally, a recent Swedish register-based study confirmed the increased cancer risk in patients with CHD [15]. This study found that the risk of cancer in CHD was 23% higher compared to age- and sex-matched controls and remained 18% higher even when patients with syndromes and organ transplant recipients were excluded. Importantly, younger patients (0–17 years of age) and those born in later cohorts (1990–2017) had more than a twofold increased total cancer risk, particularly of lymphoid or hematopoietic origin, compared with controls [15]. Furthermore, the risk of cancer in children with CHD who underwent cardiac surgery during the first year of life was almost twofold higher than in controls, supporting the notion that thymectomy or damage to the thymus gland by sternotomy during cardiac surgery may be responsible for the increased risk of cancer [15,16].

## 4. Genetic Etiology

To date, however, the potential mechanisms underlying the relationship between CHD and cancer remain poorly understood, and shared genetic anomalies may be possible factors [17].

Indeed, the dysregulation of many developmental genes may play a role in both heart development and the etiology of childhood cancer [18]. A genetic origin is also supported by the evidence that patients with chromosomal anomalies and genetic mutations manifest CHD and an increased risk of childhood cancer [19]. For instance, children with Down syndrome have a high risk for CHD and for childhood leukemia, but not for solid tumors [20].

Congenital cardiac defects, such as interrupted aortic arch, truncus arteriosus, and tetralogy of Fallot, are often present in patients with DiGeorge/velocardiofacial syndromes with chromosome 22q11.2 deletion [21]. These subjects also have an increased risk of cancer, although the mechanism behind this risk is not fully understood [22].

Germline mutations of PTPN11 are causative of Noonan syndrome, which is an autosomal dominant disorder characterized by short stature, typical craniofacial dysmorphism, skeletal anomalies, congenital heart defects, and predisposition to malignant tumors [23,24].

Interestingly, a recent study has shown that the presence of rare loss-of-function (LoF) variants in cancer risk genes was highest among patients with CHD as compared to control participants, indicating potentially shared disease mechanisms between the congenital defect and oncogenesis [25]. The authors speculate that cancer risk is increased because the germline variant provides the first of two hits needed for cancer to emerge [26]. Additional and amplifying factors, such as high lifetime radiation dose, may increase somatic variants that complement CHD LoF variants [25].

Although this hypothesis is highly speculative, children with CHD may be described as a high-risk population already having the first genetic “hit”, and, consequently, may have increased sensitivity to ionizing radiation from birth or earlier.

## 5. Radiation Exposure and Cancer Risk in CHD

Increasing exposure to low doses of ionizing radiation from intensive cardiac imaging during infancy for accurate diagnosis and effective intervention has raised concerns about the risk of cancer [27,28]. Patients with CHD are exposed to high cumulative radiation doses since they often undergo the fluoroscopically guided diagnostic and interventional catheterization and electrophysiology procedures during their lifetime [29,30,31,32].

Catheter-based fluoroscopy procedures have an effective dose in the range of 5–30 mSv, equivalent to a radiation dose of 250–1500 chest X-rays, as shown in Table 2.

However, radiation doses can vary significantly in pediatric interventional procedures depending on the patient’s age/size and the type and complexity of the procedure [28,29,30,31,32].

Moreover, children with complex heart defects often need to undergo repeated complex procedures during their lifetime, resulting in a high cumulative radiation dose [27,29].

Among pediatric cardiology patients, three types of procedures were found to be responsible for about 95% of the total collective effective dose: diagnostic catheterization, interventional catheterization, and computed tomography [29].

At the age of 15–20 years, patients with CHD already have a cumulative lifetime exposure of 20–40 mSv, corresponding to 1000–2000 chest X-rays [29].

With cumulative radiation exposure, patients acquire increasing risks of developing cancer during their lifetime [27].

Children are especially vulnerable to the oncogenic effects of radiation, and the risk of radiation-induced cancer is three to four times higher in children than in adults for a given radiation dose [33]. This is because children have a large number of more rapidly dividing cells and a longer life expectancy to express cancer risk [34].

Several epidemiological studies have been conducted to assess the risk of cancer in children exposed to radiation during cardiac catheterization procedures [35,36,37,38,39,40,41], yielding conflicting and inconclusive results (Table 3).

In particular, a retrospective cohort study based on 4891 children with CHD who underwent cardiac catheterization at a major children’s hospital in Toronto, Canada between 1946 and 1968 did not find a significant increase in leukemia or other tumors during an average follow-up period of 13 years [35,36].

A study of 674 children in Israel following cardiac catheterization between the years 1950–1970 reported a 2.3-fold excess cancer risk, with the excess mainly due to the higher incidence of lymphoma and melanoma at a mean follow-up of 28.6 years [37].

A large population-based study of 24,833 adult patients with CHD reported a significant association between ionizing exposure from cardiac procedures and incident cancer, with a possible dose-related response in adult patients with CHD who had a lifelong disease [38].

Harbron et al. [39] reported that transplantation seems to be a large contributor to elevated cancer rates in a cohort of 11,270 individuals who underwent cardiac catheterizations while aged ≤ 22 years in the UK as compared to the general population, likely due to associated immunosuppression.

Cardiac catheterization in 2770 infants (7.8% with trisomy 21) under one year of age between January 1980 and December 1998 was associated with a significantly increased cancer risk (SIR = 4.4, 95% CI: 2.5–7.2, *p* <0.001) as compared to the risk of cancer in the general German population as assessed by the German Childhood Cancer Registry [40].

Recently, the COCCINELLE study in a cohort of 17,000 children who underwent cardiac interventional procedures from 1 January 2000 to 31 December 2013, before the age of 16, found significantly higher incidences of all cancer, leukemia, lymphoma, and solid cancers as compared to the general population [41].

However, large and well-designed epidemiological studies are still needed to directly quantify the risk of cancer from exposure to doses of radiation of 10 mSv or less—the typical dose range delivered by diagnostic X-rays [42]. Other limitations and potential biases of the epidemiological studies are the lack of a complete radiation dose assessment, the presence of confounding factors (psychosocial dysfunctions, socioeconomic status, environmental exposure, and lifestyle habits, such as smoking and alcohol use), and the short duration of follow-up.

In the future, the ongoing large-cohort HARMONIC study involving a combined cohort size ten times larger than any previous epidemiological analysis should contribute to improving our understanding of the potential long-term cancer risks in CHD children [43].

The HARMONIC study is also bridged by a biological component, which will investigate biomarkers of radiation exposure and predictors of adverse effects as a complementary strategy to the traditional analytical epidemiology [43].

## 6. Molecular Epidemiology and Biomarkers for an Early Biological Effect

Molecular epidemiology was introduced in the study of cancer in the early 1980s as a complement and improvement to traditional epidemiologic approaches by tracking a continuum of events between exposure and disease [44]. With the rapid development in molecular biology and genetic methods, it is now possible to use biomarkers or surrogate endpoints able to detect early changes caused by the exposure and to identify individuals with a particularly high risk of cancer development [45]. For instance, there is now solid evidence that the high number of chromosome aberrations (CAs) in human lymphocytes predict the long-term incidence of cancer [46,47]. Interestingly, a pioneering study conducted in 1978 showed a significant increase in CAs after cardiac catheterization in children as compared to the baseline value [48]. In more recent years, several studies reported that cardiac catheterization procedures increased acute [29] and long-term chromosomal aberrations in peripheral lymphocytes [49,50]. Importantly, the mean levels of CA frequencies were also significantly higher in exposed patients compared to both healthy age- and sex-matched subjects and newborns with similar heart defects without any radiation exposure [49]. Additionally, adult patients showed shorter telomere length than control subjects [51], which may result in chromosome fusion and lead to the genome instability recognized as the cornerstone for carcinogenesis [52].

A dose-dependent increase in radiation-induced double-strand breaks (DSBs) was detected in blood samples after pediatric cardiac catheterizations by using the analysis of γ-H2AX foci in lymphocytes, resulting in risk estimates much higher than expected from the linear-no-threshold hypothesis [53]. It is important to underline that DSBs are the most serious form of DNA damage because they are difficult to accurately repair. At the cellular level, damaged DNA that is not accurately repaired results in mutagenesis and chromosomal rearrangements, which lead to genomic instability [54].

**Figure 1:**
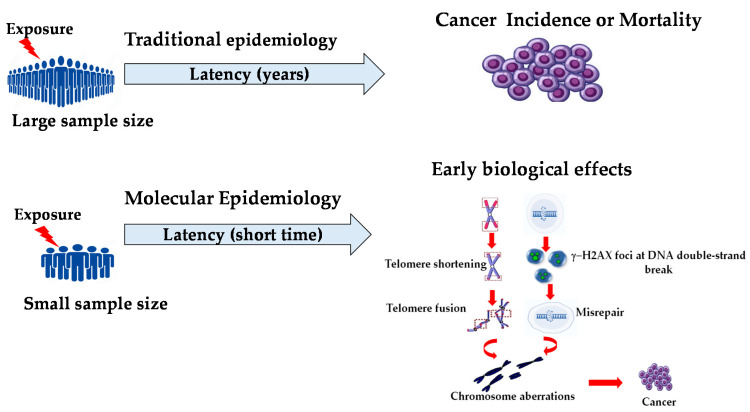
Molecular epidemiology may overcome some major limitations of traditional epidemiology and provide insights into disease causation. Validated biomarkers of effects can help to delineate the continuum of events between exposure and resulting cancer by identifying early changes in the natural history of cancer process.

Therefore, the use of biomarkers of early effects may reduce the time gap between exposure and recognition of cancer, as well as define new and more effective strategies to reduce risks, such as health surveillance and personalized risk assessment (Figure 1).

## 7. Conclusions and Future Directions

**Figure 2:**
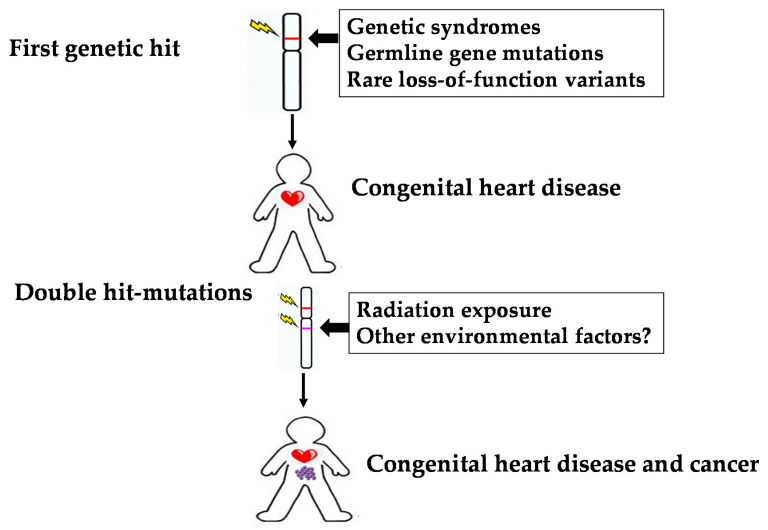
Illustration of unifying model for understanding the relationship between CHD, radiation exposure, and cancer. This model supports the notion that germline variations in CHD increase the susceptibility of induced DNA mutations.

The current level of knowledge indicates that children are a vulnerable patient population requiring particular attention for increased cancer risk. The cause of this association is multifactorial, and the interactions between genetics and ionizing radiation may be relevant in determining the risk of developing cancer (Figure 2).

The joint effect can be summarized in the following sentence: “genetics loads the gun and the radiation pulls the trigger”. However, it is likely that other environmental exposures (e.g., pollution, endocrine-disrupting chemicals) beyond ionizing radiation may be related to both CHD and cancer late in life [55,56].

At the present time, there are as yet no clear recommendations about the effectiveness of appropriate screening procedures to detect cancer in CHD patients. However, there are important strategies that can be implemented to potentially increase surveillance and timely diagnosis and decrease risk. For example, cardiologists and primary care physicians should promote patient education regarding the importance of health maintenance by providing recommendations about diet, physical activity, and modifiable risk factors, as well as strongly encouraging greater attention to cancer screening for patients with CHD. Additionally, every effort should be made to minimize exposure to low-dose ionizing radiation by using noninvasive testing with magnetic resonance imaging and echocardiography when possible or by adopting protocols with the lowest possible doses of ionizing radiation needed for addressing the specific clinical question.

Furthermore, it is critical to continue conducting research on this topic to gain a greater insight into how genetic background and risk factors interact to initiate cancer. This information may be useful for improving surveillance with screening protocols and preventive measures able to protect the most vulnerable patients.

Future research should also investigate whether CHD patients with specific genetic disorders (e.g., Down Syndrome, deletion of 22q11) that predispose them to a high risk of cancer are hypersensitive to the biological effects of ionizing radiation exposure from cardiac catheterizations, as reported by previous in vitro evidence on cell lines from patients after X-irradiation [57].

Other biological studies can be planned to determine whether the use of a near-zero radiation approach can significantly abrogate the radiation-induced DNA damage as compared with the conventional fluoroscopic technique [58].

Finally, epidemiological studies should be integrated with research with longitudinal measurements of biomarkers of risk and susceptibility in large cohorts of patients with long-term prospective follow-up [43]. These new studies will generate the evidence needed to optimize treatment in CHD patients and develop better radiation protection to reduce cancer and late radiation-induced toxicities.

## Figures and Tables

**Table 1:** Previous studies on the association between CHD and cancer.

Study	Subjects	Design	Results
Olsen M, et al., 2014, [11]	15,905 CHD children/young born from 1977 to 2008; 801 with Down syndrome or Fanconi anemia. Data sources: Danish National Registry of Patients and Danish Cancer Registry.	Register-based study.	Increased cancer risk in CHD cohort (SIR 1.63, 95%CI: 1.22–2.13). No association was found by excluding patients with Down syndrome or Fanconi anemia.
Lee YS, et al., 2015, [12]	31,961 children/young adults, newly diagnosed from 1998 to 2006. Data sources: Taiwan National Health Insurance Research Database.	Population-based cohort study.	Increased cancer risk in CHD (SIR 1.45, 95%CI 1.25–1.67), particularly hematologic, CNS, and head/neck malignancies. Age and chronic liver disease are independent risk factors for cancer occurrence.
Gurvitz M, et al., 2016, [14]	34,965 CHD adults alive in 2005. Data sources: Quebec Congenital Heart Disease Database from 1983 to 2005.	Population-based cohort study.	Twofold increased cancer prevalence in CHD compared to general population. Breast, colorectal, and uterine are the most common types for women; prostate, colorectal, and bladder for men.
Collins II RT, et al., 2018, [9]	65,585 children with structural birth defects born from 1988 to 2004; 25,981 with CHD but without chromosomal anomalies. Data sources: California Birth Defects Monitoring Program Registry and California Cancer Registry.	Population-based cohort study.	Increased cancer risk in CHD compared to non-CHD (HR 2.6, 95% CI 1.9–3.6). Lymphoma is more than 8 times higher in CHD and correlated with disease complexity.
Mandalenakis Z, et al. 2019, [13]	21,982 CHD and 219,816 without CHD born from 1970 to 1993. Data sources: Swedish Patient Register.	Registry-based prospective cohort study.	Increased cancer risk in CHD compared to controls (HR 2.24, 95% CI 2.01–2.48), higher among CHD from the most recent birth cohort.
Karazisi C, et al., 2022, [15]	89,542 CHD and 890,472 controls without CHD, born between 1930 and 2017. Data sources: Swedish Health Registers and Swedish Total Population Register.	Register-based study.	The overall cancer risk is 23% higher in CHD compared to controls and 18% higher excluding those with syndromes and organ transplant recipients. The highest cancer risk was found in CHD cohort aged 0–17 years.
Kampitsi et al., 2022, [10]	4,178,722 children born between 1973 and 2014; 66,892 CHD subjects. Data sources: Swedish Medical Birth Register, National Patient Register, and Swedish Cancer Register.	Population-based cohort study.	Increased risks of lymphomas and hepatoblastomas in CHD, even excluding subjects with Down syndrome. Stronger association was observed in complex CHD.

*CHD*, congenital heart diseases; *HR*, hazard ratio; *SIR*, standardized incident ratio.

**Table 2:** Average radiation dose for common ionizing procedures in CHD patients.

Procedure	Effective Dose (mSv)	Equivalent CXRs
Chest X-ray	0.02	1
Diagnostic catheterization	6.0	300
Patent ductus arteriosus occlusion	7.6	360
Closure of atrial septal defect	2.8	280
Aortic coarctation	6.8	340
Balloon valvuloplasty	8.1	410
Electrophysiological study	3.2	160
Regular PM or ICD implant	4	200
Ablation procedure	15.2	760

From references [28,29,30,31,32]. *CXRs*, chest X-rays; *mSv*, millisievert.

**Table 3:** Previous studies on the association among CHD, cancer, and radiation exposure.

Study	Subjects	Design	Exposure Assessment	Results
Spengler RF, et al., 1983, [35]	4891 CHD children assessed by CC during 1946 to 1968. Data sources: records from the Hospital for Sick Children in Toronto and Ontario cancer death file.	A retrospective cohort study.	Estimation of radiation exposure per CC.	No excess of cancer mortality in children who underwent CC.
McLaughlin JR, et al., 1993, [36]	3915 children < 18 years at the time of procedure who underwent CC between 1950–1965. Data sources: records from Ontario Hospital for Sick Children and Ontario Cancer registry.	Monocenter retrospective cohort study.	Number of procedures. Time period of the first CC per child.	No risk of cancer among the cohort and no association between exposure and increased cancer risk were found.
Modan B, et al., 2000, [37]	674 children with congenital anomalies who underwent CC between 1950–1970. Data sources: records from 3 major Israeli medical centers and the National Cancer Registry.	Multicenter retrospective cohort study.	Number of procedures.	Increased risk of all cancers (SIR = 2.3; 95% CI: 1.2–4.1); lymphomas the most relevant. No dose–response association was observed.
Harbron RW, et al., 2018, [39]	11,270 CHD patients who underwent CC before 22 years. Data sources: UK hospital records, NHS Central Register, NHS Transplant Registry.	Multicenter retrospective cohort study.	Number of CC and CT procedures. Estimated cumulative organ doses for CC and CT procedures.	Higher cancer rate in CHD compared to general population was found, especially in transplant recipients. The number of CC/CT or organ doses was associated with post-transplant cases.
Cohen S, et al., 2018, [38]	24,833 CHD adult patients (18 to 64 years); 602 cancer cases. Patients with genetic disorders were excluded. Data sources: Quebec Congenital Heart Disease Database.	Retrospective, population-based cohort and control–case study.	Cumulative LDIR exposure.	Cumulative cancer incidence in CHD was 15.3% (95% CI, 14.2–16.5). Cases had more LDIR-related cardiac procedures than controls, and cumulative LDIR exposure was associated with cancer.
Stern H, et al., 2020, [40]	2770 CHD children who underwent CC under 1 year of age between 1980–1998. Data sources: hospital database and German Childhood Cancer Registry.	Retrospective single-center observational study.	Effective radiation doses.	Increased cancer risk (SIR 4.4 95%CI: 2.5–7.2) in CHD in the first year of life. No significant association was found between cancers and effective radiation doses.
Abalo KD, et al., 2021, [41]	17,104 CHD patients (<16 years) at first CC between 2000–2013. Data sources: Patient cohort comes from 15 France hospitals and the National Childhood Cancer Registry.	Multicenter retrospective cohort study.	Number of CC procedures.	Increased SIRs in CHD for all-cancer, leukemia, lymphoma, and solid cancers compared to general population. No difference in number of procedures between cancer and non-cancer cases were observed.

*CHD*, congenital heart diseases; *CC*, cardiac catheterization; *SIR*, standardized incidence ratio; *NHS*, national health service; *CT*, computed tomography; *LDIR*, low-dose ionizing radiation.

## Data Availability

Not applicable.
